# Identification of New Structural Fragments for the Design of Lactate Dehydrogenase A Inhibitors

**Published:** 2016

**Authors:** D.K. Nilov, A.V. Kulikov, E.A. Prokhorova, V.K. Švedas

**Affiliations:** Belozersky Institute of Physicochemical Biology, Lomonosov Moscow State University, Lenin Hills 1 , bldg. 40, Moscow, 119991, Russia; Faculty of Fundamental Medicine, Lomonosov Moscow State University, Lomonosov prospect 31- 5 , Moscow, 119192, Russia; Faculty of Bioengineering and Bioinformatics, Lomonosov Moscow State University, Lenin Hills 1, bldg. 73, Moscow, 119991, Russia

**Keywords:** Lactate dehydrogenase, inhibitor, sulfo group, sulfonates, molecular modeling, docking

## Abstract

Human lactate dehydrogenase A plays an important role in the glucose metabolism of tumor cells and constitutes an attractive target for chemotherapy. Molecular fragments able to bind in the active site of this enzyme and form hydrogen bonds with the Arg168 guanidinium group, as well as additional interactions with the loop 96–111 in the closed conformation, have been identified by virtual screening of sulfonates and experimental testing of their inhibitory effect. The sulfo group can occupy a similar position as the carboxyl group of the substrate and its structural analogs, whereas the benzothiazole group attached via a linker can be located in the coenzyme (NADH) binding site. Thus, the value of merging individual structural elements of the inhibitor by a linker was demonstrated and ways of further structural modification for the design of more effective inhibitors of lactate dehydrogenase A were established.

## INTRODUCTION

**Fig. 1:**
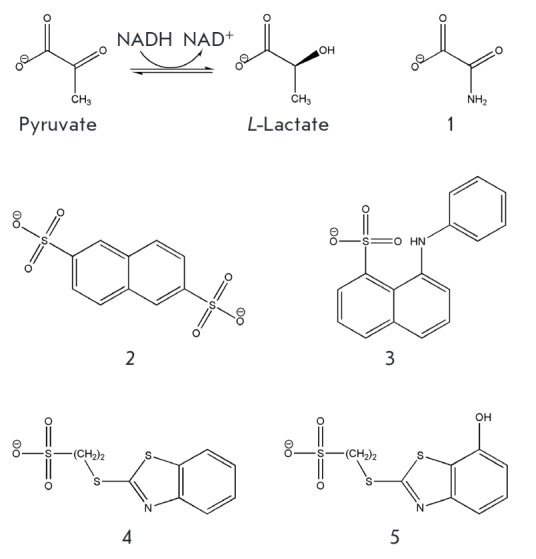
Chemical structures of human LDH-A substrates and inhibitors (**1**-**5**). **1 **– oxamate, **2 **– naphthalene-2,6- disulfonate, **3 **– 8-(phenylamino)naphthalene-1-sulfonate,** 4 **– 2-(benzothiazol-2-ylsulfanyl)-ethanesulfonate, **5 **– 2-(7-hydroxybenzothiazol-2-ylsulfanyl)-ethanesulfonate.

Lactate dehydrogenase (LDH) catalyzes the conversion of the glycolysis product pyruvate to lactate, accompanied by the oxidation of NADH to NAD^+^ (Fig. 1). In a healthy human organism, LDH isoform A (LDH-A) is found primarily in skeletal muscles; isoform B – in heart muscle; and C – in testes [1, 2]. In many tumor cells, activation of pyruvate conversion by LDH and reduced pyruvate oxidation in the mitochondria is observed. This alteration of metabolism is known as the Warburg effect [3, 4]. One of the reasons for the elevated glycolysis is an increased expression of LDH-A [5, 6]. This enzyme is an attractive oncological target as it plays an important role in the viability and proliferation of tumor cells [7-9]. Therefore, a search for selective inhibitors of human LDH-A and investigation of their effects at the cellular level are of particular interest.

Several classes of LDH-A inhibitors are described in the literature [10, 11], and most of them contain a carboxyl group. Oxamate (the structural analog of the substrate) and its numerous derivatives can serve as an example [12, 13]. Hydrogen bonds of the carboxyl group with a conserved Arg168 residue are crucial for the binding of pyruvate and oxamate [14, 15]. Residues of the mobile loop 96–111 [16] also participate in the binding of the substrate, coenzyme and inhibitors, among which the role of Arg105 should be emphasized (it stabilizes the transition state in the course of substrate conversion). The crystal structures of human LDH-A complexes, where the loop 96–111 is either in the closed or open conformation depending on the structure of the inhibitor, have been determined [17-19]. In the development of LDH-A inhibitors, an attempt has been made to find compounds able to interact with both the substrate and coenzyme binding sites [20, 21]. A promising way to solve this problem may be a search for molecule fragments – small molecules capable of forming specific interactions with selected protein regions. Being subsequently connected by a suitable linker, these fragments may serve as a basis for new and more effective inhibitors of the enzyme. The analysis of published data on the binding of the substrate, as well as oxamic and malonic acid derivatives, points to the importance of electrostatic interactions with the Arg168 guanidinium group in the active site of LDH-A. Given this fact, the goal was set to explore the possibility of using a negatively charged sulfo group in the design of the structure of new inhibitors.

Sulfo-substituted derivatives of naphthalene 2 and 3 (*Fig. 1*) were referred to in work devoted to the search for inhibitors of LDH from the parasitic microorganism* Plasmodium falciparum *that causes malaria. However, they were found to exert only a weak inhibitory effect [22]. The crystal structure of LDH from *P. falciparum* in a complex with **2 **(PDB ID 1u4s) revealed that the sulfo group of the inhibitor interacts with Arg171 (it corresponds to Arg168 in the human LDH-A). The authors assumed that inhibitor **2 **binds in a similar manner to the apo form and the LDH-coenzyme complex without competing with NADH. It should be noted that there are significant differences in the arrangement of the active site in human and parasite LDHs, mainly associated with the position of the coenzyme and mobile active site loop, which is 5 residues shorter in the human LDH [23]. This suggests that sulfonate-based structural fragments of human LDH-A inhibitors should differ from compounds **2 **and **3**. In our previous work, we constructed models of human LDH-A for searching for inhibitors competing with the substrate and coenzyme, and also determined the structural criteria for the selection of potential inhibitors [24]. The developed approach was used for the screening of molecular fragments with a sulfo group which might be additive components in the design of more effective inhibitors of LDH-A.

## EXPERIMENTAL SECTION

Virtual screening for LDH-**A **inhibitors was performed among low-molecular-weight compounds from the Vitas-M library [25]. Using the ACD/Spectrus DB 14.0 software [26], compounds containing a sulfo group and conforming to the rule of three [27, 28] were retrieved from the library. This rule defines the ranges of physicochemical parameters associated with molecule fragments (molecular weight < 300, log *P *≤ 3, hydrogen bond donors ≤ 3, hydrogen bond acceptors ≤ 3, and rotatable bonds ≤ 3). Molecular docking of compounds from the obtained focused library was performed using Lead Finder 1.1.15 in the “extra precision” mode [29, 30] and the models of human LDH-A (with and without the bound molecule of NADH) constructed in our previous work [24]. At the first step of the selection of inhibitors, some compounds were eliminated when the distance between the sulfur of the sulfo group and the guanidinium carbon of Arg168 at their binding with LDH-A exceeded 5.5 A. The remaining compounds that fitted the criteria of the structural filtration were tested for their ability to form hydrogen bonds and hydrophobic contacts with residues of the loop 96–111 [24]. Visualization and analysis of the structures were performed using VMD 1.9.2 [31].

Experimental measurement of enzyme activity was conducted using LDH from rabbit muscle (Sigma-Aldrich). Potassium phosphate buffer 0.1 M, pH 7.0 was used for the preparation of the solutions and performance of the measurements. An enzyme solution containing 1% (g/ml) bovine serum albumin (BSA) was prepared immediately prior to the measurements. The LDH-A activity was monitored spectrophotometrically at 340 nm using a Shimadzu UV-1800 spectrophotometer by detecting the decrease in the NADH absorbance at the conversion of pyruvate to lactate. The reaction mixture containing the buffer, pyruvate (400 μM), NADH (20 μM), and an inhibitor was placed into the cuvette, thermostated for 5 min at 37°C, and then the reaction was started, adding an aliquot of the enzyme. The initial rate of the enzyme-catalyzed reaction was determined in two independent experiments. The IC_50_ value (concentration of an inhibitor at which the enzyme activity is reduced by 50%) was determined by varying the concentration of an inhibitor from 0 to 8 mM.

## RESULTS AND DISCUSSION

Crystallographic studies revealed that sulfo-substituted derivative **2 **is capable of binding only to the open conformation of LDH from *P. falciparum *in which the active site loop is disordered. Obviously, the structural fragments containing a sulfo group and capable of binding to the enzyme in the closed conformation, i.e. when effective interaction with the loop 96–111 is expected, should substantially differ from compounds **2** and **3**. To identify new fragments, a set of sulfonic acids and their salts (71 compounds) was selected from a library of low-molecular-weight compounds. Compounds were docked into the active site of the previously developed models of human LDH-A, and then their ability to mediate a significant electrostatic interaction with the Arg168 residue, as well as additional interactions with the loop 96-111 in the closed conformation, was analyzed. The most promising inhibitor, compound **4**, capable of efficient binding with the apo form of LDH-A (Δ*G*^calc^ = –9.9 kcal/mol), was chosen as a result of screening.

The inhibitory properties of compound **4 **were experimentally tested against LDH from rabbit muscle, whose active site has high structural similarity with that of human LDH-A [32]. The IC_50_ value was determined to be 1.2 mM. Interestingly, inhibitor 4 binds in a similar manner to the earlier investigated oxamate derivative STK381370 (ΔGcalc = –7.9 kcal/mol, IC_50_ 5 mM) [24], forming additional interactions with the loop 96–111. However, the interaction of 4 is more efficient. The results indicate that the earlier developed model of the closed enzyme conformation adequately simulates the binding of compounds of various classes.

**Fig. 2:**
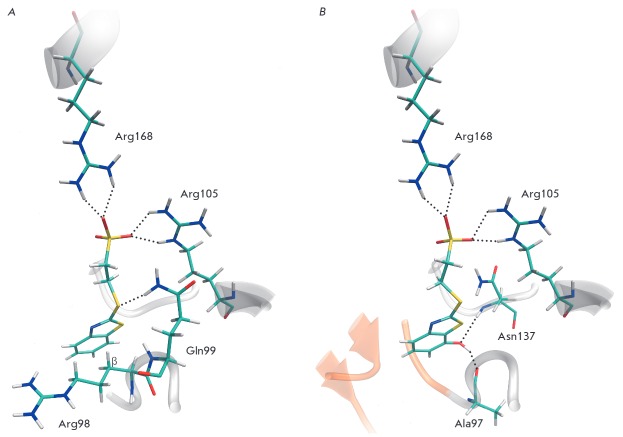
Positions of inhibitors in the active site of human LDH-A revealed by molecular modeling. A – Binding of compound** 4**: hydrogen bonds of a sulfo group with Arg168 are shown, as well as interactions with the active site loop residues Arg98, Gln99, and Arg105. *B *– Binding of compound **5**: additional hydrogen bonds of hydroxyl substituent with Ala97 and Asn137 are shown, interactions with Arg98 and Gln99 are not depicted. A region occupied by the adenine moiety of the coenzyme is colored orange.

Localization of the structural fragment with the charged sulfo group in the substrate binding site leads to the stabilization of the inhibitor’s position due to the formation of hydrogen bonds with guanidinium groups of Arg168 and Arg105 (*Fig. 2A*). A very important issue in the design of LDH-A inhibitors is the way of connecting individual elements in the structure. So, for example, interaction of compound **4 **with both the substrate binding site and that of NADH’s nicotinamide nucleotide is possible owing to the flexibility of a linker between the sulfo and benzothiazole groups. The thioether linker forms a hydrogen bond with the side chain of Gln99, while the benzothiazole group, located in the site of the first ribose residue of the coenzyme, forms a favorable hydrophobic contact with the Cβ-atom of the Arg98 side chain. It should be noted that the abovementioned interactions with the residues Arg98, Gln99, and Arg105 important for the stabilization of the active site loop take place when the closed conformation is formed. There are also additional interactions at the sulfonate binding: formation typical for oxamate hydrogen bonds between the sulfo group, Asn137, and Thr247, hydrophobic contacts of the linker with Ile241 and benzothiazole group with Val30, hydrogen bond of a ring’s heteroatom with the Asn137 carboxamide (not shown in the figure).

Among the sulfo derivatives examined in the course of screening, there were structures without a flexible linker (including naphthalene derivatives), with a linker elongated by one methylene unit, and with benzene, pyrrole, and pyridine replacing benzothiazole in compound **4**. All of them were characterized by a lower binding energy and were incapable of forming interactions sufficient for the stabilization of the loop 96-111 in the closed conformation. This indicates that scaffold **4 **is optimal for binding in the active site and may serve as a basic structure for further modifications. For example, the introduction of the hydroxyl group at position 7 allows this substituent to occupy the site responsible for binding of the 3’-OH group of the first ribose residue of NADH and to form hydrogen bonds with the Ala97 and Asn137 backbones (compound **5**, *Fig. 2B*). The value of the calculated binding energy (Δ*G*^calc^ = –10.9 kcal/mol) shows that this modification leads to an additional energy gain. Increased efficiency of enzyme inhibition due to the introduction of substituents into the benzothiazole group seems to be a promising perspective for the further merging of structural fragments aimed at achieving additive (and perhaps synergistic) effects in the development of novel LDH-A inhibitors.

## CONCLUSIONS

The aim of the present study was to select new molecular fragments for the design of LDH-A inhibitors able to form interactions of a charged acid group with Arg168 and amino acid residues of the active site loop in the substrate binding site, as well as interactions with the coenzyme binding site typical of substrates and previously described inhibitors. As a result of virtual screening and experimental validation of inhibitory properties, new fragments have been identified that comprise a sulfo group, linker, and benzothiazole group. The performed study allowed us to uncover the most important interactions and the amino acid residues that stabilize the position of inhibitors containing a sulfo group (Ala97, Arg98, Gln99, Arg105, Arg168) in the closed enzyme conformation. Thus, the methodology for LDH-A inhibitors search has been tested and ways for further optimizing inhibitor structures have been outlined.

## Abbreviations

LDH: lactate dehydrogenase
LDH-A: lactate dehydrogenase isoform A
